# Reconceptualizing Emotion Recognition Ability

**DOI:** 10.3390/jintelligence11060123

**Published:** 2023-06-19

**Authors:** Konstantinos Kafetsios, Ursula Hess

**Affiliations:** 1 School of Psychology, Aristotle University of Thessaloniki, 54124 Thessaloniki, Greece; 2 Psychology Department, Palacký University, 779 00 Olomouc, Czech Republic; 3 Institute of Psychology, Humboldt University, 10117 Berlin, Germany; ursula.hess@hu-berlin.de

**Keywords:** EI abilities, emotion perception, accuracy, bias

## Abstract

Emotion decoding accuracy (EDA) plays a central role within the emotional intelligence (EI) ability model. The EI-ability perspective typically assumes personality antecedents and social outcomes of EI abilities, yet, traditionally, there has been very limited research to support this contention. The present paper argues that the way in which EDA has been conceptualized and operationalized in EI research has ignored developments in social perception theory and research. These developments point, on one hand, to the importance of embedding emotion expressions in a social context and, on the other, to reformulating the definitions of emotion decoding accuracy. The present paper outlines the importance of context in the framework of a truth and bias model of the social perception of emotions (Assessment of Contextualized Emotions, ACE) for EI abilities.

“Imagine a situation in which a young man visits a friend in the hospital who has been in a car accident. The first area of emotional intelligence involves perceiving emotions. As the young man surveys the hospital room, the visiting relatives, and his unconscious friend, he may wonder, ‘What is each family member feeling?’ Perhaps he perceives the worry and anxiety in their faces. Feelings are complex; also emerging from within him may be fear of his own mortality, and a guilty relief—with a surge in energy—in response to being spared the accident himself and remaining unharmed.”

(Mayer et al. 2008, American Psychologist, p. 506)

## 1. Introduction

As the opening statement indicates, perceiving emotions is key to many aspects of our everyday social life. Most interactions—even trivial ones—are tinged by emotion. Whether it is a salesperson who tries to convey their enthusiasm for a product, a loved one who is complaining about their problems, or a person who is visiting a friend in the hospital, emotions have an important role in everyday human communication. Therefore, emotion decoding accuracy (EDA), the accurate identification of emotions, plays a vital role in regulating personal and social relationships (Manstead et al. 1999). It facilitates coordination with others, enhances communication in general, and serves as a crucial element of the “affective glue” that binds individuals in dyadic interactions (Feldman et al. 1991; Niedenthal and Brauer 2012). Emotions can manifest through various channels, including voice, body posture, gestures (e.g., Bänziger et al. 2009), and tactile cues (Hertenstein et al. 2006). However, facial expressions are among the most extensively researched sources of emotional communication. 

In this vein, the ability to perceive and understand the (facial) emotion expressions of others is a core social skill in the emotional intelligence framework (Salovey and Mayer 1989–1990). Emotional intelligence (EI) is conceptualized as a set of cognitive abilities involved in monitoring one’s own and others’ emotions, cognitively discriminating among emotions, and using emotions in thinking and behavior (Mayer et al. 2008). The concept of emotional intelligence, initially broadly popularized (Goleman 1995), has captivated the scientific imagination of social scientists for more than three decades since its inception (Salovey and Mayer 1989–1990). At the theoretical level, EI reflects the extent to which a person attends to, processes, and acts on information of an emotional nature, intrapersonally and interpersonally. As such, a key facet of the EI concept has been its social dimension.

EI theorists (see Matthews et al. 2007) generally cite Thorndike as one among the first to acknowledge the existence of a form of social intelligence, specifically, “the ability to understand and manage men and women, boys and girls—to act wisely in human relations” (Thorndike 1920, p. 228). However, with notable exceptions (e.g., Lopes et al. 2004; Moeller et al. 2014), this theoretical conjecture remained largely untested as the EI literature has taken a predominantly intraindividual differences approach while downplaying the interpersonal and social dimensions of EI abilities. As we will explain later, this criticism applies more than anything else to EDA and emotion recognition abilities, which is the focus of this article. 

The present paper reviews the current approach to the study of emotion perception within the EI-related literature. We argue that the way that EDA has been conceptualized and operationalized within EI abilities research has downplayed the social dimension and social functions of EDA and related EI abilities and ignored developments in social perception theory and research. These developments point, on one hand, to the importance of showing emotion expressions in a social context and, on the other, to the need to reformulate the definitions of emotion decoding accuracy and inaccuracy. The present paper outlines the relevance of context in the framework of a truth and bias model of EDA. In doing so, we will emphasize research on facial expressions of emotions in humans. However, it should be noted that the basic points we are making regarding a contextualized assessment of emotion expressions are presumed by us to be equally applicable to other emotion communication channels.

## 2. Emotion Perception as Part of EI Abilities Set

Emotion perception is a fundamental human capacity for at least one additional, theoretical, reason. It is the key emotional ability upon which other emotional abilities (those under the emotional intelligence remit (emotion facilitation, understanding, and management) are thought to be built (Joseph and Newman 2010; Mayer and Salovey 1997). Emotion perception is generally defined as the ability to attend to and cognitively process the emotion expressed by another person verbally, facially, with the body, or by using a combination of these modalities (Elfenbein and MacCann 2017; Olderbak and Wilhelm 2017). Emotion perception ability has been suggested to have evolutionary roots and affinities with empathic and emotion communication processes (e.g., Buck 1984) and has been described as the most basic of the skills that constitute emotional intelligence (Salovey and Grewal 2005). As defined by Mayer and Salovey’s (1997) ability model, emotion perception refers to “the ability to identify emotions in oneself and others, as well as in other stimuli, including voices, stories, music, and works of art” (Brackett et al. 2006, p. 781). 

Although the EI ability model takes a broader stance on emotion perception by including the basic ability of registering emotional stimuli in self and others (Joseph and Newman 2010), in this article we will focus on the part of emotion perception ability that concerns the facial emotion expressions of others. As such, EDA is related to, although distinct from, cognate but broader constructs such as interpersonal sensitivity, defined as “accuracy in noticing and recalling another’s nonverbal cues, speech content, or physical appearance” (Hall et al. 2009, p. 150), emotional competence (Saarni 1999) or affective social competence, the sending and receiving of affective messages and experiencing affect (Halberstadt et al. 2001), or social intelligence more broadly (Weis and Süß 2007).

As an ability, EDA is a specific skill, part of a broader set of social cognitive abilities related to inferring psychological states from social perception.^1^ Elfenbein and MacCann (2017) point to Carroll’s (1993, p. 4) definition of an ability as “some kind of performance, or potential for performance with a clear end performance criterion.” Applying Carroll’s definition to EDA, it is crucial to clarify what the performance criterion is in each case. In most assessments of EDA, such as the Faces parts of the Diagnostic Assessment of Non-Verbal Abilities (DANVA, Nowicki and Duke 2001), the Profile of Nonverbal Sensitivity (PONS, Rosenthal et al. 1979), the Multi-modal Emotion Recognition Test (MERT, Bänziger et al. 2009), the Geneva Emotion Recognition Test (GERT, Schlegel et al. 2014), and the Japanese and Caucasian Brief Affect Recognition test (JACBART, Matsumoto et al. 2000), or the Reading the Mind in the Eyes Test (Baron-Cohen et al. 2001), there is a pre-determined ‘ground truth’ criterion. In the case of the Mayer—Salovey–Caruso Emotional Intelligence Test (MSCEIT, Mayer et al. 2003) Faces Perception Test the criterion is either an ‘expert judgment’ or a culture-level consensual agreement within a stimulus set.

For these and other assessments of EDA, participants are usually presented with contextless, prototypical facial expressions drawn from standardized sets of facial expressions (PAF, Ekman and Friesen 1976; KDEF, Lundqvist et al. 1998; ERI, Scherer and Scherer 2011), or with (facial) recognition tasks (DANVA, Nowicki and Duke 2001; JACBART, Matsumoto et al. 2000; PONS, Rosenthal et al. 1979; GER [faces], Schlegel and Mortillaro 2019); MSCEIT [faces], Mayer et al. 2003). Typically, participants are required to select from a list of emotion labels the one that best describes the depicted emotional expression. The label is considered accurate when it matches with the researcher-determined label. That is, decoding accuracy is usually defined as the ability to associate one (correct) label with a single emotion expression shown without social context. Notably, the MSCEIT [faces] part allows for multiple emotions to be indicated. 

As such, the typical measurement procedure does not engage participants’ social competences and ignores the important impact of context for emotion recognition (Barrett et al. 2011; Hess and Hareli 2016). A second important drawback of this approach is that the underlying definition of what constitutes accuracy in decoding emotion is limited. In what follows we will outline the importance of these two aspects—the inclusion of context and the definition of accuracy for a conceptualization of EDA that is useful for the prediction of real-life social outcomes. 

## 3. Two Ways to Decode Emotions

In our view, the fundamental problem with traditional approaches to measuring EDA is the (often implicit) assumption regarding how people decode expressions. Specifically, the tests assume that pattern matching is the only relevant underlying process. Pattern matching associates specific features of the expression with specific emotions (Buck 1984). For example, upturned corners of the mouth or lowered eyebrows are recognized as smiles or frowns, respectively, and a perceiver can, thus, conclude that the individual is happy or angry. This assumption then justifies that participants are presented with contextless faces, often even with hairlines removed, to better show-cast these informative elements. The perceiver’s task is to match a label to a perceived constellation of features without consideration of the context and expresser. This process can be conceived of as a cognitive task that does not rely on the perceiver’s wider social knowledge but only on knowledge about specific facial configurations, similar to the approach used by facial expression recognition software.

Specifically, there is a second process, which is based on the perceiver’s social knowledge: perspective taking. Perspective taking can be used to justify an observed expression after the fact, such as when we try to explain to ourselves why a friend flew into a rage at a seemingly innoxious comment but can also be used to deduce the likely expression of someone who experiences an event. For example, learning that someone received good news allows the prediction that the person is now happy rather than disappointed. Another source of information is the social group membership of the expresser. People hold stereotypes about members of different groups and these stereotypes can inform emotion perception (Kirouac and Hess 1999). We propose that, in most situations, observers use this form of perspective taking and their accumulated emotion knowledge to actively make sense of the expression in its context. Such a process involves social knowledge engagement. That is, to be able to use pattern matching to deduce emotional states based on facial expressive information in context, participants engage in social cognition and use theory of mind.

In this vein, a recent study (Antypa et al. 2023) demonstrated that emotion expressions that are presented in a social context, together with the use of scalar judgments, activated brain regions associated with theory of mind and social information processing, whereas the process of applying single labels to contextless stimuli did not. Notably, the target of the task was always the same person, showing the same expression; what differed was the presence of others in the image and the type of rating task. Only the perception of expressions in a group-embedded setting was associated with extended brain activations, in accordance with evidence from social cognition research (Arioli et al. 2021). 

We do not claim that people never use the cognitive puzzle approach in real life—they very much do, for example, when pointing out expressive features in a picture, such as a sympathetic smile or an ironic look; however, we claim that perspective taking is ubiquitous in everyday social contexts.

## 4. What Is Emotion Decoding Accuracy? A Truth and Bias Model

Accepting that there are two different processes involved in EDA (pattern matching and perspective taking) leads to a second important issue—how to define accuracy. This question seems simple and straightforward at first glance, but how one defines accuracy has far reaching consequences for the conclusions one can draw (Funder 1995; Kruglanski 1989; Zaki and Ochsner 2011). The general problem with any performance-based measure is the establishment of the correct answer or ground truth (Funder 1995). For emotion expressions there are several options. For example, a label can be derived from the expressive parameters for a given prototypical emotion described by Ekman and Friesen (1976). Alternatively, a label could be derived from the emotion the expresser felt during the expression (Levenson et al. 1991). The MSCEIT proposes two criteria to establish the correct answer: judgments by experts and the consensus of participants from a given culture (see also Mayer et al. 2003). However, in all these cases accuracy is based on the notion that there is one and only one correct answer. That is, emotion expression is presumed to reflect a single “pure” emotion within a given cultural context (even if the specific label may vary by culture) and that the decoders are accurate when they are able to decode that given expression. 

We contend that the assumption that a single emotion label adequately describes an emotion expression is problematic. First, it is not certain that in the abovementioned methods the portrayed expressions are “pure” representations of a given emotional state. Second, even if one assumes that a test succeeds in capturing “pure” emotions, there is good evidence that these “pure emotion” stimuli would not be perceived as such. Specifically, observers tend to perceive multiple emotions even when judging emotional expressions considered to be “pure” (Russell and Fehr 1987; Yrizarry et al. 1998). These mixed perceptions may be based on different sources such as facial morphology. For example, Hess et al. (2012) showed that, amongst other factors, the wrinkles and folds in older faces add to the mixed perception of “pure” expressions. Another source of mixed perception is linked to personality. For example, individuals with more insecure attachment tend to over-attribute negative affect to peoples’ facial displays (Magai et al. 2000). Therefore, it is unlikely that a single label adequately captures perception even when “pure” emotion expressions are used in emotion recognition tests. This is even more of an issue in everyday interactions in which more subtle and ambiguous expressions are used that are more open to interpretation (Ekman 2003; Motley and Camden 1988) and, consequently, require more sense-making efforts.

Moreover, when people choose only one label out of several, only one form of inaccuracy can be assessed: mistaking one emotion for another. This approach has been criticized by Lyusin and Ovsyannikova (2016) who suggest the use of a multidimensional response format or scalar rating scales where participants are asked to indicate all the emotions they can discern in an expression (see also, Matsumoto 2005). Scalar rating scales can better capture the actual perception process by allowing the observers to describe emotions as mixed rather than pure. Unlike the misclassification of emotions in a constrained choice task, this type of inaccuracy does not inevitably lead to a tradeoff where greater accuracy equates to reduced inaccuracy.

We argue that the ability to accurately perceive “secondary” emotions, which we refer to as “bias”, is theoretically independent from the ability to accurately perceive the target emotion. That is, the fact that someone perceives some level of sadness in an expression that is primarily considered to express anger does not have to influence the perception of anger. However, in this case, the fact that sadness is also identified is very relevant as there are good reasons why this tendency should show a link to individual differences as we will outline below.

We interpret accuracy and bias as defined above, in line with the truth and bias model of social perception (West and Kenny 2011). This model posits accuracy and bias in social perception as two theoretically distinct processes; bias is considered to arise from systematic factors that influence perception and is distinct from error. Furthermore, both bias and accuracy serve a social purpose. Thus, bias, the perception of secondary emotions, is not simply the opposite of making accurate judgments about the target emotion. Instead, biased perception (secondary emotions) and accurate perception of the main emotion signal can be seen as two dimensions that coexist and impact emotion perception (Kenny 2011). 

## 5. How ‘Social’ Is EI—Emotion Perception Ability? 

The second important issue in traditional EDA approaches has been the neglect of social context. Critically, the EI ability approach has considered emotion perception from an intraindividual perspective, neglecting the social context in which emotion perception takes place. We contend that this neglect of context explains why the evidence base for the social correlates of EDA from an abilities perspective is thin. 

Notably, even though some studies that take a broader, personality-based approach around trait or mixed models of EI (Petrides and Furnham 2003) find that self-reported emotion perception ability is related to more socially supportive relationships with friends and family members (e.g., Ciarrochi et al. 2001), for the most part the evidence that emotion perception ability has real-world consequences is far from overwhelming.

Much of the supporting evidence for social correlates of EDA comes from the organizational behavior literature and mostly using methods related to but distinct from the emotion perception task of the MSCEIT. Rubin et al. (2005) found leaders’ performance on the DANVA predicted transformational leadership behavior at a moderate level. In a negotiation study simulating undergraduate buyers and sellers (Elfenbein et al. 2007), emotion perception accuracy was measured using the Singapore Picture Scale, a test similar to the JACBART (Matsumoto et al. 2000). Better emotion perception on the part of sellers increased the amount of money gained overall by the negotiating pair and was marginally related to the proportion of money received by the seller individually. Buyers’ emotional perception showed no effect. Further, emotion recognition capacity measured using a version of the GERT (Schlegel et al. 2014) was positively related with both peer status and friendship quality in Chinese primary school children (Wang et al. 2019), thereby providing evidence of its interrelatedness with the interpersonal interactions of children. 

Further, evidence for a relationship between emotion recognition ability and personality traits with presumed relevance for social interaction skills is very sparse. Agreeableness, a prosocial personality trait, was associated with employees’ higher scores on the MSCEIT faces scale, especially for persons with higher power (Côté et al. 2011, study 3). Prosocial traits, such as Social Value Orientation (SVA, Murphy et al. 2011) showed a limited, nonsignificant association with an EDA task, the identification of emotion expression from composite faces (Kaltwasser et al. 2017). The poverty of this research record, given not only the theoretical arguments but also the definite face validity of the notion that EDA should be somehow related to social outcomes, is a clear sign of a problem in measuring the underlying concepts.

## 6. The Role of Context

As noted above, we posit that in most everyday situations people use perspective taking to understand the emotions of others. This process depends on rich stimuli that allow people to perceive the expresser in a social context. Although it is widely understood that emotion perception rarely works context-free in real life (Barrett and Kensinger 2010; Hess and Hareli 2018), emotion perception research has typically used context-free facial expressions as stimuli. Even more surprisingly, emotion research has largely ignored the most common form of context we experience in everyday life: other people (Matsumoto and Sung Hwang 2010). Because emotions usually occur in social (real or imagined) interactions, the presence of other people is a feature that is common to many emotion-provoking eliciting contexts. Yet, the presence of other people has mainly been considered from a cultural perspective (e.g., Kafetsios and Hess 2015; Masuda et al. 2008), when in fact it is a ubiquitous element of everyday interaction. The facial expressions of bystanders to an event can influence how the event itself is perceived (Hess and Hareli 2018), and the facial reactions of recipients of an expression can affect the meaning attributed to the expression (Hareli and David 2017).

Presenting participants with emotion expressions shown by a group of individuals provides an important and very relevant “social framing” for the EDA task. This social framing promotes the use of perspective taking which, in turn, infuses the perception process with “biases” that reflect the personality and values of the perceiver. In this sense, “biases” are not to be equated with errors as they constitute an expression of the perceiver’s social cognition and personality. This point will be discussed in more detail below.

## 7. A Social Cognitive Model of Decoding Emotion Expressions

To summarize, we contend that emotion perception is based on multiple sources of information, including the expression displayed, contextual cues, and the observer’s social schemas (Hess and Hareli 2016). In real-life situations, the perception of emotions rarely occurs in isolation from contextual factors (Hess and Hareli 2016). In complex situations where the social perception of more than one emotion is plausible, we can expect people to also perceive more than one emotion. In traditional EDA research these additional emotions are considered to be “noise”—the use of context-free minimalistic expressions devoid of even hairline was an effort to reduce this “noise”. However, we argue that the tendency to inaccurately perceive bias in the form of “secondary” emotions is theoretically independent of the accurate perception of the signal, which is the target emotion (West and Kenny 2011). Both accuracy and bias can have independent and meaningful implications for interpersonal interactions (Kenny and Acitelli 2001). For example, a person with low signal perception may misinterpret the emotional state of the other person, leading to inappropriate reactions that irritate the angry person. In contrast, a person who shows both high accuracy and high bias may correctly perceive the anger but also perceive it to be influenced by additional emotions such as sadness or disgust, and in reacting to these perceived emotions may create a somewhat strained and uncomfortable interaction.

The truth and bias model (West and Kenny 2011) highlights the importance of considering both accuracy and bias in research on emotion perception. This model suggests that bias results from systematic factors that influence perception and both bias and accuracy have social functionality that can be empirically tested. Based on these considerations we propose the Assessment of Contextualized Emotion (ACE, Hess et al. 2016; Kafetsios and Hess 2013, 2015, 2022) as new approach to EDA. We contend that this approach can establish deeper connections between social cognition and accuracy processes, as proposed over ten years ago by Zaki and Ochsner (2011).

## 8. The ACE Model for EDA

The Assessment of Contextualized Emotion (ACE) situates expressers within the context of other individuals. Specifically, participants see a central expresser surrounded by two other individuals who were filmed during an interaction. Their task was to narrate an event that elicited a given emotion (anger, happiness, sadness, disgust) and which they had experienced together. This type of activity typically elicits the narrated emotion (Rimé 2009). From these interactions, still frames were selected from groups that reported having felt the emotion during the narration. 

**Figure 1:**
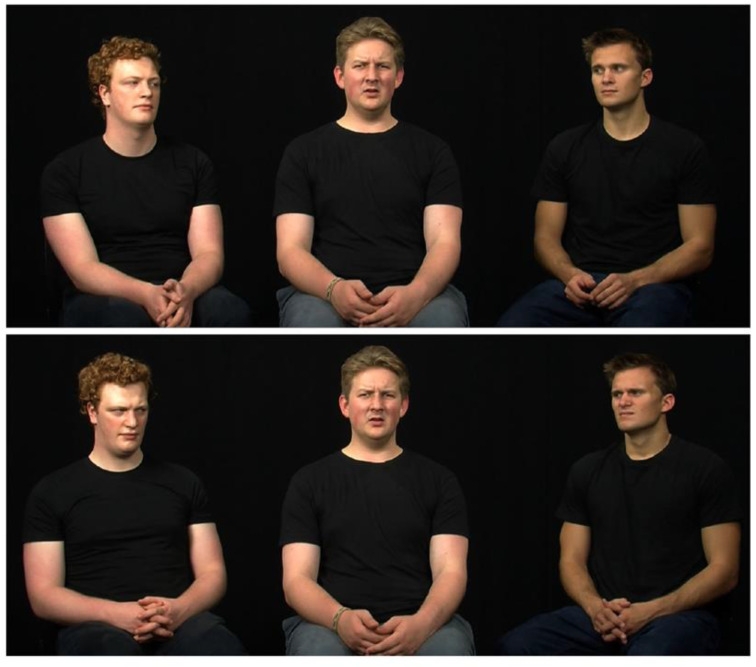
Example from the ACE stimuli set.

**Figure 2:**
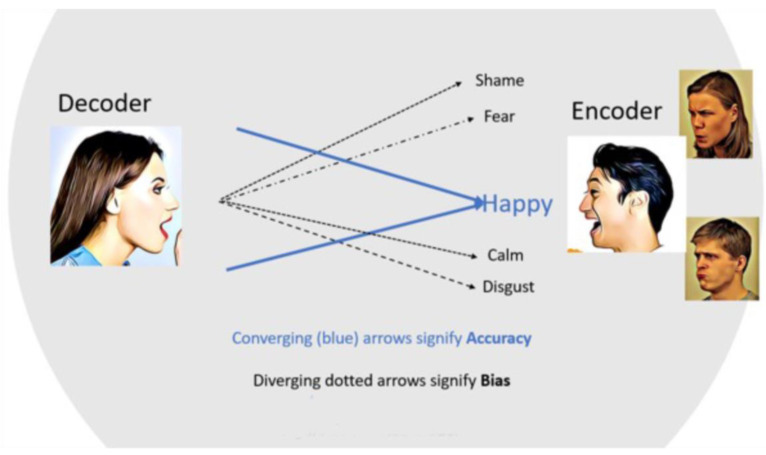
Graphical depiction of the ACE model.

The stills were modified such that two individuals in the periphery express congruent or incongruent emotions with respect to the central person’s expressions that are to be decoded. Typically, the presence of others is a common contextual element that primes social processing modes. Observers rate the intensity of those expressions on an emotion profile, using several dimensional scales to indicate the intensity of a series of emotions, some of which do not correspond to the depicted emotions by the central character (see Figure 1). ACE accuracy is the average rated intensity of emotion shown by the central person, whereas bias is the average intensity of all other emotion scales (see Figure 2). Perceived intensity is a valid indicator of accuracy and the low-to-mid intensity expressions selected correspond well to spontaneous real-life expressions (Hess et al. 1997).

Thus, the ACE method creates an assessment context that permits the differentiation between accurate evaluation of the presented focal emotions (accuracy) and the simultaneous evaluation of nonpresented, secondary emotions (bias). As is demonstrated in the next section, accuracy and bias can be considered as largely independent EDA dimensions. Additional information about the ACE model and stimuli can be found in Hess and Kafetsios (2021) and Kafetsios and Hess (2022), and the ACE stimuli can be obtained upon request from the authors.

## 9. Contextualized Emotion Perception and Its Social, Personality, and Cultural Correlates

Several studies from our laboratories have shown that ACE accuracy and bias have unique, measurable, and meaningful effects for social interaction. A first set of three studies, two conducted in Greece and one in Germany (Hess et al. 2016), provided initial evidence for a link between ACE measures and indices of everyday social interaction quality. Participants completed the ACE task in the laboratory and then participated in an event sampling study focused on all meaningful dyadic interactions over a 10-day period. ACE accuracy and bias predicted self-reported parameters of interaction quality, whereas MSCEIT faces (Mayer et al. 2003) did not. Specifically, ACE accuracy in Greece was associated with higher social interaction quality indicators for interactions with close others (partners, close friends, or family), whereas ACE bias was associated with lower social interaction quality, especially within close relationships. In Germany, higher ACE accuracy was associated with all social interaction quality indicators across levels of intimacy (Hess et al. 2016). Importantly, ACE accuracy and bias were unique predictors of social interaction quality. The unique effects of ACE accuracy and bias on social interaction quality imply that one can be simultaneously both accurate and biased, which is in line with the truth and bias model of social perception (West and Kenny 2011). 

In a more recent study (Kafetsios and Hess 2019), ACE bias was associated with alexithymia, the difficulty in identifying and describing emotions, and both alexithymia and ACE bias contributed to problems in everyday dyadic interactions and relationships. Participants completed the Toronto Alexithymia Scale (TAS) and the ACE task in a laboratory session, followed by a 10-day event sampling study on the quality of their naturally occurring social interactions. The Difficulties in Identifying Feelings (DIF) subscale of the TAS was negatively related to all indices of quality of social interaction, and DIF was positively and moderately strongly correlated with bias. Importantly, ACE bias was found to mediate the effects of DIF on social interaction outcomes.

These results suggest that bias as measured in the ACE task can tap into the lack of attunement in dyadic social interactions observed in people with alexithymia. Such a lack of attunement in everyday social interactions should also influence wellbeing. Indeed, Kafetsios et al. (2023a) have documented in two studies that ACE accuracy contributes to overall wellbeing through the quality of social interaction. These findings highlight the importance of considering contextualized measures of emotional functioning in understanding social interaction and wellbeing.

Incidentally, several of the above studies, which used different versions of the ACE task, have provided consistent evidence that the standard way to assess emotion perception ability in the EI framework, the MSCEIT face part, is negatively related to ACE bias and not related to ACE accuracy. In three studies, two in Greece and one in Germany, the MSCEIT face part was inversely related to ACE cartoons and ACE faces bias (*r*(165) = −.44, *p* < .01; *r*(84) = −.50, *p* < .001; *r*(122) = −.48, *p* < .001, respectively; Hess et al. 2016 studies 1, 2, and 3). In a larger study with Greek participants (Kafetsios and Hess 2022, Study 7), the MSCEIT faces part was negatively associated with bias assessed by a short version of the ACE faces, β = −.71, *p* < .001. In none of these studies were the MSCEIT faces scores significantly related to the ACE accuracy scores. MSCEIT faces scores also failed to predict the quality of social interaction (Hess et al. 2016) as well as prosocial personality traits (Kafetsios and Hess 2022).

This is a remarkably consistent pattern of results that largely informs our understanding of the nature of the ACE model. It stands to reason that the MSCEIT faces and the ACE bias tap into more stereotypical, culturally shared biases in emotion decoding. A big part of EI and emotion perception is based on emotional knowledge (Izard 2001) and this emotional knowledge can vary as a result of culture or personality differences. 

Another set of studies looked at the prosocial personality characteristics associated with ACE accuracy and bias. In seven studies conducted in two laboratories in Greece and Germany (Kafetsios and Hess 2022), we tested relationships between the ACE and personality traits that tap into the social domain. ACE accuracy was associated with more emotion reappraisal, less emotion suppression, less loneliness, and higher wellbeing. In turn, ACE bias was associated with less emotion reappraisal, more insecure attachment, and a more interdependent self-construal. Importantly, a traditional hit rates approach (associating one correct label to a single emotion expression) did not show the same associations. 

The results for insecure attachment were partly replicated in a large sample of 2240 participants from 12 different cultures (Kafetsios et al. 2023b) who completed the short version of the ACE and the Experiences in Close Relationships (Fraley et al. 2000), a standard self-report measure of adult attachment organization. Anxious attachment was associated with both more accuracy and more bias, whereas avoidant attachment was associated with less accuracy and more bias. Importantly, neither avoidance nor anxiety were associated with EDA assessed via classic hit rates. That is, associating one correct label to a single emotion expression did not provide the same information as the contextualized assessment of emotions in terms of accuracy and bias. These results speak to the independence of accuracy and bias in line with the truth and bias model (West and Kenny 2011). 

Lastly, using an early version of the ACE task, results from two experimental studies in Greece (Kafetsios and Hess 2013, 2015) suggest that chronic and temporarily raised independent self-construal increased accuracy in the ACE task. This effect is primarily understood in social–cognitive terms: because independent self-construal (chronic or naturally varying) is associated less with interdependent self-construal with more attention to context (Masuda et al. 2008), more interdependent observers are more likely to integrate perceptions of the surrounding faces into their judgment and, thereby, increase bias.

Based on the above, we also expected that higher social class will be associated with higher accuracy in the ACE task because higher SES is associated with a more independent self-construal (Miyamoto et al. 2018). This is because higher social class individuals are considered to focus more on the self, whereas lower class individuals tend to pay more attention to the social context. In the aforementioned recent multiculture study (12 cultures N = 2440, Kafetsios et al. 2023a) in Europe, Northern America, and Southern and Eastern Asia participants completed a self-construal scale (Singelis 1994), and the MacArthur Subjective Social Status Scale (SSS, Kafetsios et al. 2023a). SSS was found to be associated with higher accuracy in decoding emotions (but not less bias) and this association was mediated by independent self-construal. Parental education level, an objective index of social class, was associated with less bias.

## 10. Conclusions 

In this article, we present a critique of the standard ability approach to emotion decoding accuracy (EDA). This approach, which relies on tests that use prototypical faces out of context, fails to capture the nuances of everyday social interaction skills. We argue that the conceptualization and operationalization of EDA in emotional intelligence research have not kept pace with advancements in social perception theory and research. These advancements highlight the significance of social context in assessing EDA and redefine the meaning of accuracy and bias in EDA within the framework of a truth and bias model of the social perception of emotions. This approach emphasizes the usefulness of accuracy in social emotion perception and its adaptive social value, as demonstrated by ACE accuracy and bias’s ability to predict various social functionality correlates. As such, we consider the ACE to be a better-suited alternative to the use of the MSCEIT faces for EI research interested in the interpersonal sequalae of emotion recognition ability.

In the above cited research, we have started to address links between ACE assessed EDA and personality on one hand and some aspects of social interaction quality on the other. However, much of this research involved simple questionnaires and cross-sectional samples. Additionally, the developmental aspect of EDA was completely neglected. This implies a rich field for future research that considers assessments of personality through peer ratings and longitudinal assessments. Conversely, the use of state measures of personality and of observed interactions in the laboratory can allow for a more fine-grained analysis of the relationship between personality, EDA, and interaction behavior. Furthermore, the ACE focuses only on facial expressions and uses still frames. Future versions should use dynamic (video) stimuli and the inclusion of other channels. In short, using the ACE model to develop more refined tests and applying these to the wide field of social interactions opens a rich avenue of potential research.

## Note

1 The evidence for the internal consistency across emotion perception abilities is mixed. On the one hand, as Elfenbein and MacCann (2017) note, different emotion perception tasks positively relate to other facets of EI, such as emotion understanding (see Ferguson and Austin 2010; Libbrecht and Lievens 2012; Vonk et al. 2015). On the other hand, Joseph and Newman’s (2010) meta-analysis found only moderate evidence for a link between emotion perception and intelligence (ρ = .10). Yet, this link is one of Mayer and Salovey’s (1997) criteria for justifying EI as an intelligence.
